# Author Correction: Chlorine activated stacking fault removal mechanism in thin film CdTe solar cells: the missing piece

**DOI:** 10.1038/s41467-022-28059-4

**Published:** 2022-01-14

**Authors:** Peter Hatton, Michael J. Watts, Ali Abbas, John M. Walls, Roger Smith, Pooja Goddard

**Affiliations:** 1 Department of Mathematical Sciences, Loughborough University, Loughborough, Leicestershire UK; 2 Department of Chemistry, Loughborough University, Loughborough, Leicestershire UK; 3 Centre for Renewable Energy Systems Technology (CREST), Loughborough University, Loughborough, Leicestershire UK

**Keywords:** Devices for energy harvesting, Solar cells

Correction to: *Nature Communications* 10.1038/s41467-021-25063-y, published online 23 August 2021.

The original version of this Article omitted the Acknowledgements: EPSRC Studentship 1801035; EPSRC Grant Nos. EP/P020232/1, EP/L000202, EP/R029431, EP/P020194.

This has been corrected in both the PDF and HTML versions of the Article.

